# Selective-area growth of single-crystal wurtzite GaN nanorods on SiO_x_/Si(001) substrates by reactive magnetron sputter epitaxy exhibiting single-mode lasing

**DOI:** 10.1038/s41598-017-12702-y

**Published:** 2017-10-05

**Authors:** Elena Alexandra Serban, Justinas Palisaitis, Chia-Cheng Yeh, Hsu-Cheng Hsu, Yu-Lin Tsai, Hao-Chung Kuo, Muhammad Junaid, Lars Hultman, Per Ola Åke Persson, Jens Birch, Ching-Lien Hsiao

**Affiliations:** 1 Thin Film Physics Division, Department of Physics, Chemistry, and Biology (IFM), Linköping University, SE-581 83 Linköping, Sweden; 2 Department of Photonics, National Cheng Kung University, Tainan, 701 Taiwan; 3 Department of Photonics and Institute of Electro-optical Engineering, National Chiao-Tung University, Hsinchu, Taiwan

## Abstract

Selective-area growth (SAG) of single-crystal wurtzite GaN nanorods (NRs) directly onto Si(001) substrates with un-etched native SiO_x_ amorphous layer, assisted by a patterning TiN_x_ mask fabricated by nanosphere lithography (NSL), has been realized by reactive magnetron sputter epitaxy (MSE). The GaN NRs were grown vertically to the substrate surface with the growth direction along *c*-axis in the well-defined nano-opening areas. A 5-step structural and morphological evolution of the SAG NRs observed at different sputtering times depicts a comprehensive growth model, listed in sequence as: formation of a polycrystalline wetting layer, predominating *c*-axis oriented nucleation, coarsening and coalescence of multi-islands, single NR evolution, and finally quasi-equilibrium crystal shape formation. Room-temperature cathodoluminescence spectroscopy shows a strong GaN bandedge emission with a uniform luminescence across the NRs, indicating that the SAG NRs are grown with high quality and purity. In addition, single-longitudinal-mode lasing, attributed to well-faceted NR geometry forming a Fabry–Pérot cavity, was achieved by optical pumping, paving a way for fabricating high-performance laser optoelectronics using MSE.

## Introduction

The selective-area growth (SAG) of nanorods (NRs), by the use of patterned substrates, presents the advantages of resultant NRs with uniform size leading to uniform properties, but also site-specific controlled growth^1–5^. For example, it was previously shown that an inhomogeneous size distribution of GaN NRs results in a broadening of the photoluminescence (PL) signal^6^ or the dependency of the photocurrent generation efficiency in the NR photodetectors on the NR diameter^7^. Moreover, since the use of SAG gives control over the position of the NRs, this enables deeper studies of adatom kinetics and mobility, making it possible to define the explicit growth mechanism^5^. Due to the strong influence of the geometry and dimension on the physical properties of nano-size materials, a detailed understanding of the growth mechanisms is required, especially for further exploitation of such a versatile method as magnetron sputter epitaxy (MSE) for industrial device production.

The NR geometry allows for an increased photon extraction efficiency^1^, and the use of SAG NRs has been successfully demonstrated in the fabrication of single color and monolithic integration of red-green-blue (RGB) high-brightness light-emitting diodes (LEDs)^2–4^. From the device perspective, an ordered array characterized by uniformity in sizes and shapes is desired in high-density integration and chip processing technology. Among others, this could be implemented in the replacement of conventional planar LEDs in the solid-state lighting market. Other applications are in the fields of nanoelectronics^8^, nanosensing^9^ or photovoltaics^10^.

In addition, semiconductor laser, fabricated by low-dimensional NRs, has high potential to be integrated with very large scale integration circuits, which can be used for many novel applications in image scanning, optical communication, information processing, optical interconnects, data storage *etc*.^11,12^. The semiconductor-based nanolasers can provide both a gain medium and a cavity for lasing. The lasing mechanism in a laser diode is determined by the geometry of the optical cavity. Single-mode lasing happens when the stimulated emission of coherent light is contained within a thin cavity. If the waveguide is wide compared to the wavelength of light, then multiple transverse optical modes will contribute to the lasing. Most common optical cavities, such as the Fabry–Pérot (FP) cavity, whispering-gallery modes (WGMs), and nanophotonic crystals, often require well-faceted NR surfaces and/or periodic NR arrays in nanolaser systems for achieving high-quality factor (Q)^13–15^.

Although plenty of research studies exist on SAG of GaN NRs by metal-organic chemical vapor deposition (MOCVD)^3,16,17^ and molecular-beam epitaxy (MBE)^2,4,18,19^, no work has been performed by MSE. The advantage of MSE is that it is a versatile technique, which opens countless possibilities when compared to the other two methods, like: easy integration on an industrial platform, reproducibility, smaller cost, and avoidance of using dangerous precursors. On the other hand, from epitaxy point of view, MSE offers the possibility to grow high-quality III-nitride epitaxial films at room temperature, thanks to enhancement of surface diffusion by the low-energy ions^20,21^. In the other cases of MBE and MOCVD processes, temperatures higher than 700 °C are often required to obtain high-quality films since the mobility of adatoms is mainly contributed from thermal energy^22,23^.

The potential of using MSE to grow high-quality GaN NRs and thin films has been presented in previous studies^24–27^. Here, we demonstrate position- and diameter-controlled GaN NRs grown on Si(001) substrates, assisted by a TiN_x_ mask patterned by NSL, employing direct current (dc)-reactive MSE using a liquid Ga target. Our MSE process enables the growth of well-faceted *c*-axis oriented GaN NRs directly onto the native SiO_x_ layer inside nano-opening areas, which was not yet reported in the literature. Our model, consisting of 5 growth stages is proposed based on the characterization by scanning electron microscopy (SEM), x-ray diffraction (XRD), transmission electron microscopy (TEM), high-resolution scanning transmission electron microscopy (STEM), and energy-dispersive x-ray spectroscopy (EDX). This shows a different growth behavior from initial nucleation to the formation of mature single NRs inside nano-openings compared to SAG NRs on isostructural templates, such as GaN and AlN, by MBE and MOCVD^2–4,16–19^. In addition, the as-grown SAG GaN NRs exhibit strong bandedge emission at room temperature and single-mode lasing performed by optical pumping, confirming the high-quality and purity of the material. Our MSE SAG of GaN NRs on Si wafers paves the way to engage into the integrated-circuit (IC) industry for large-scale fabrication of Si-based hybrid GaN optoelectronics.

## Results and Discussion

### SAG of GaN NRs

**Figure 1:**
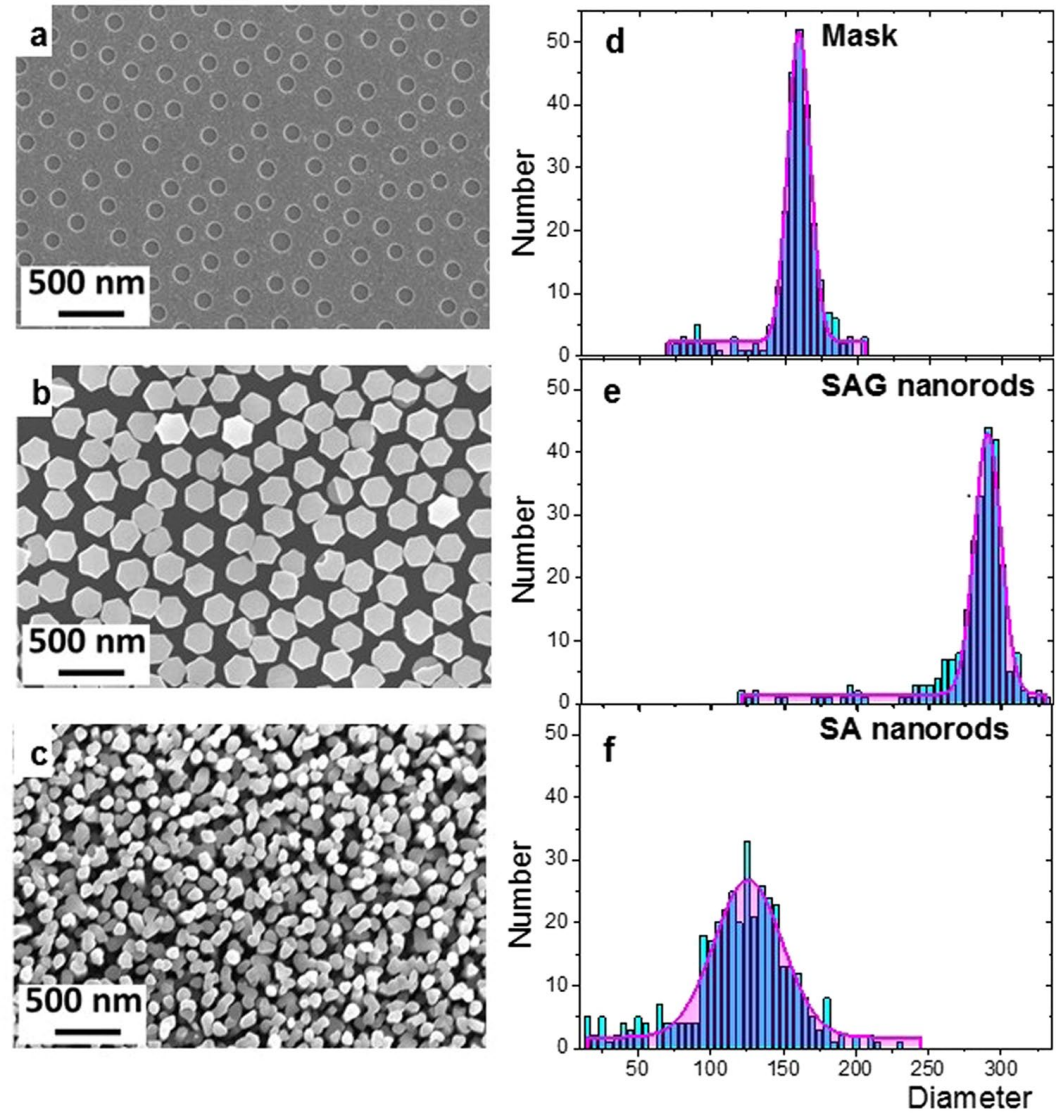
Top-view SEM micrographs and corresponding diameter distributions of: (**a**,**d**) nano-openings in the mask, (**b**,**e**) GaN NRs grown on the patterned substrates for 1 hour and (**c**,**f**) self-assembled NRs grown under identical conditions as SAG NRs.

Figure 1 shows top-view SEM micrographs of the TiN_x_ mask (Fig. 1a), SAG GaN NRs grown for 1 hour (Fig. 1b), and self-assembled (non-SAG) (Fig. 1c, from previous experiments at identical conditions). Figure 1 additionally includes the corresponding histograms of NR and opening size distributions. As can be seen from the figure, successful SAG of GaN NRs by MSE is achieved when a TiN_x_ mask is employed to the substrate surface. The size and morphology of NRs are rather homogenous, and the NRs are distributed uniformly over the entire substrate with the size of 10 × 5 mm^2^ (not shown here). In contrast, the size, density, morphology, and spatial organization of the self-assembled (SA) GaN NRs is different to the SAG NRs. However, the growth directions of both SAG and SA GaN NRs are along the *c*-axis orientation and perpendicular to the substrate surface, see Supplemental information in Figure S1.

When comparing the size distribution of the nano-openings in the mask and the diameters of the SAG NRs, a strong correlation can be observed. The nano-openings are dispersed in the range of 140 to 180 nm (with a pronounced maximum at 157 nm), while the NR diameters are found in a similarly confined interval 250–310 nm, with an average value of approximately 290 nm.

The morphology of the SAG GaN NRs reveal discrete hexagonal-like rods in contrast to the SA GaN NRs where the cross-sections are not well-defined, presumably due to the coalescence of multiple rods. The SA NRs exhibit a much smaller average diameter which is dispersed in the range of 50–200 nm, with an average of approximately 125 nm. The diameter distribution of the SA NRs is approximately 110 nm, while the diameter distribution of SAG NRs is confined to 60 nm. Additionally, the height is uniform for the SAG NRs of approximately 300 nm (not shown). In contrast, the SA NRs are characterized by a pronounced height inhomogeneity, as shown in previous studies^24–26^.

### Nucleation and time evolution of SAG-GaN NRs

**Figure 2:**
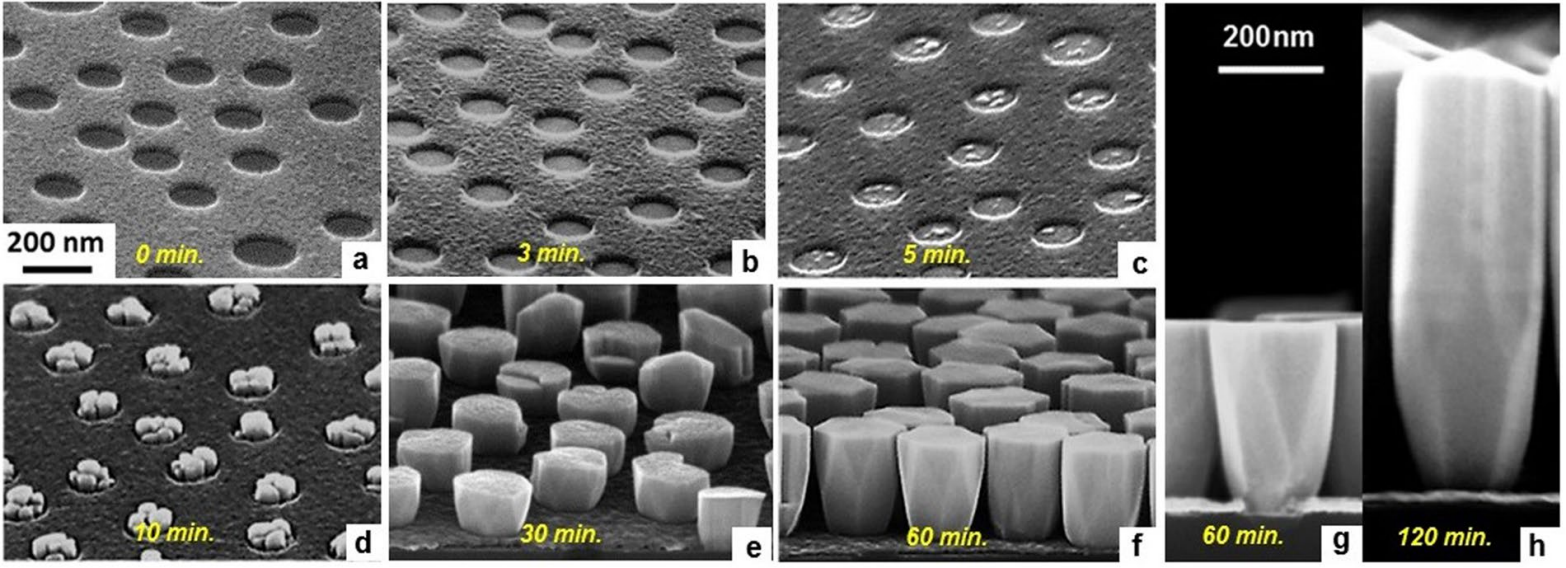
Birds-view (**a**–**f**) and side-view (**g**,**h**) SEM micrographs showing the nucleation and time evolution of the GaN NRs on the NSL patterned substrate. (**a**) The bare patterned substrate before the growth. (**b**) An initial wetting layer formed after 3 minutes. (**c**) 2D to 3D growth mode transition after 5 minutes. (**d**) Growth of the individual nuclei after 10 minutes, and (**e**) their coalescence and pattern fill after 30 minutes. (**f**,**g**) The complete coalescence of the nuclei and growth under the form of a single NR after 1 hour. (**h**) Image of a single NR after 2 hours exhibiting the specific axial growth and blunt pencil-shaped termination. Figures (**a**–**f**) and (**g**,**h**) have the same scale, respectively.

The nucleation and the time evolution of the GaN NRs are studied by analyzing samples grown for different sputtering times as shown in Fig. 2. Initially, a thin wetting layer seems to have formed inside the openings after 3 minutes growth, as can be seen from the strong contrast difference in the openings when comparing the as-prepared masked structure with the 3 minutes sample, see Fig. 2a and b. After 5 minutes growth (Fig. 2c), multiple nuclei protrude from the seed layer and are exclusively observed inside the nano-openings, representing a 2D to 3D growth mode transition. The number of nuclei formed inside an opening (typically 1–7) is found to increase with opening size and growth time, as is shown in Fig. 2c and Supplemental information S2. Mostly, around 4–5 nuclei are found in openings with size of 150–170 nm. These nuclei grow larger with time resulting in island formation, see Fig. 2d. No apparent faceting of these islands is observed at this stage. After 30 minutes of growth, the islands have coalesced into what is unmistakably the early stages of single faceted NRs approximately 100 nm in height (Fig. 2e). The surfaces are inclined with respect to the NR’s growth direction making the NR wider with time. The shape reforming behavior of coalesced NRs to achieve a quasi-equilibrium crystal shape is presumed to lower the total free surface energy^5,28^. After 1 hour of growth, the quasi-equilibrium shape is reached, see Fig. 2f. The side surfaces of NR’s upper part becomes aligned with the growth direction while the facets at the base are uncompromised, despite the continued growth. By further increasing growth time, the length of the NRs is extended, and additionally, the NR’s top preferentially develops a blunt pencil-shaped termination (Fig. 2g,h). The same top-shape is observed also in samples grown for longer times, like 4 hours (not shown here).

### Microstructural and compositional characterization

#### Nucleation and coalescence phase

**Figure 3:**
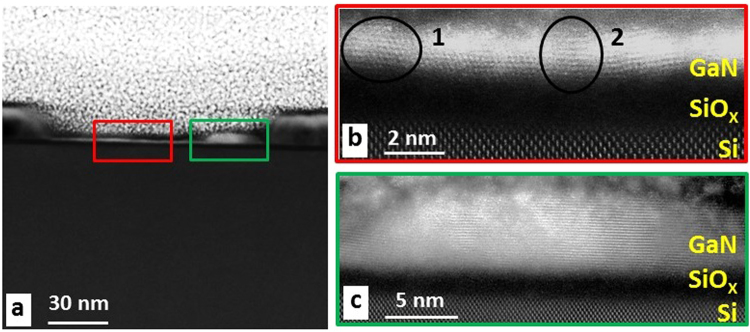
Cross-sectional HR-STEM image of the sample grown for 5 minutes. (**a**) STEM image acquired inside an opening, identifying the wetting layer and nucleus. (**b**) Enlarged view of the area marked by the red square in (**a**) identifying the polycrystalline GaN wetting layer with different orientations indicated. (**c**) Enlarged view of the area marked by the green square in (**a**) identifying the nucleus.

HR-STEM images acquired from inside one of the mask openings for the 5 minutes sample are presented in Fig. 3. Inside the opening, an apparent layer structure can be seen on the Si substrate, with a single nucleus exhibiting increased brightness, located near the edge of the opening (indicated by a green rectangle). The enlarged magnifications of the layered structure (Fig. 3b) shows that a crystalline layer was grown on a darker interlayer on the crystalline Si substrate. Both layers are ~2 nm thick. By examination of the lattice fringes from the polycrystalline layer, it is assumed to be GaN, given the spacings; 2.49 Å in region 1, and 2.58 Å in region 2, which are around half of *c* lattice constant. As to the dark interlayer, it is assumed to be the native amorphous SiO_x_ layer since no HF etching process was applied before sample growth. The apparently single crystal nucleus (Fig. 3c), with a diameter of approximately 30 nm, exhibits only one crystalline orientation with the *c*-axis oriented along the growth direction.

**Figure 4:**
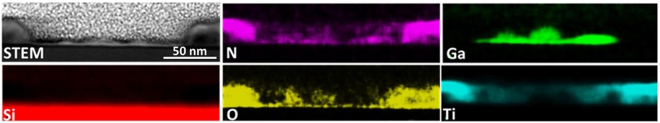
STEM-EDX elemental maps of the sample grown for 5 minutes showing an HAADF-STEM image acquired inside an opening with 3 nuclei present. The corresponding N, Ga, Si, O, and Ti elemental maps identify the 3 nuclei and the thin GaN wetting layer on the native SiO_x_ amorphous layer inside the TiN_x_ mask opening.

To confirm the elemental distribution, cross-sectional STEM-EDX elemental maps were acquired across the interface. Figure 4 shows that the wetting layer, interlayer, and surrounding mask in an opening are composed of GaN, SiO_x_, and TiN_x_, respectively. In this opening, three protruding GaN nuclei grown together with the wetting layer on the amorphous SiO_x_ layer can be observed. The oxygen signal detected in the rest of the sample is attributed to an artifact due to the damage resulted from FIB preparation procedure. The accuracy of EDX analysis can be affected also by the overlapping peaks of N, O, and Ti. EDX N mapping is not possible in TiN, due to overlap of the Ti-L peak with the N-K peak. EELS should be used in order to separate N (400 eV EELS edge) and Ti (450 eV) signal (see Supplemental information Figure S3).

The STEM-EDX results confirm that a 1–2 nm thick GaN layer was exclusively grown on the amorphous SiO_x_ interlayer inside the mask opening. The protruding nuclei above the wetting layer are oriented along *c*-axis, so that subsequent growth will be dominated by *c*-axis nuclei, with a higher growth rate than other orientations when growing on a disordered surface, as it has been proposed in a similar case of wurtzite InAlN NRs grown on amorphous carbon substrates^29^.

#### Steady-growth phase

**Figure 5:**
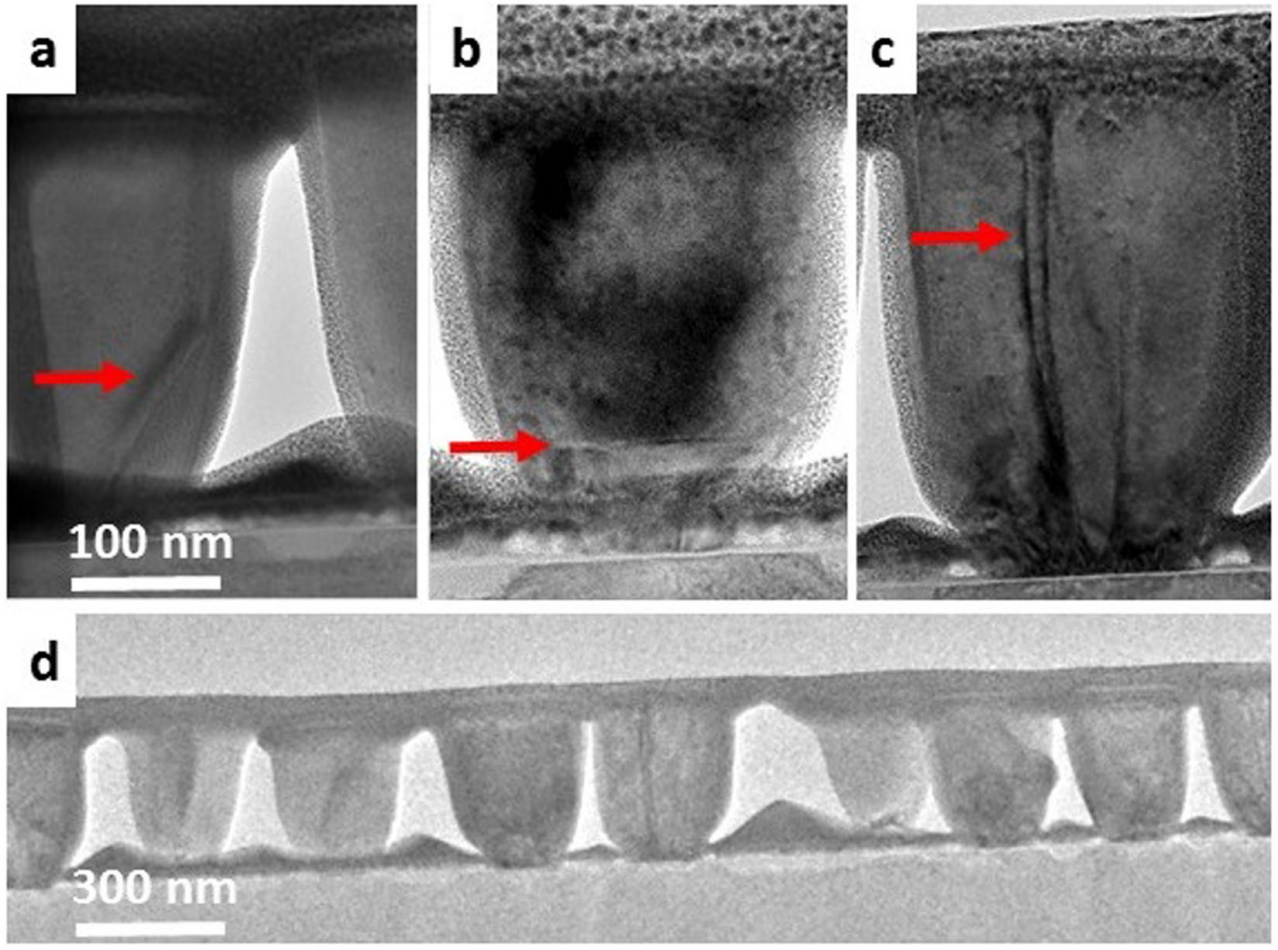
Cross-sectional TEM images of different types of NRs present in the 1 hour sample. The red arrows indicate structural defects (**a**–**c**) and (**d**), overview of the 1 hour sample, showing a small number of defects.

**Figure 6:**
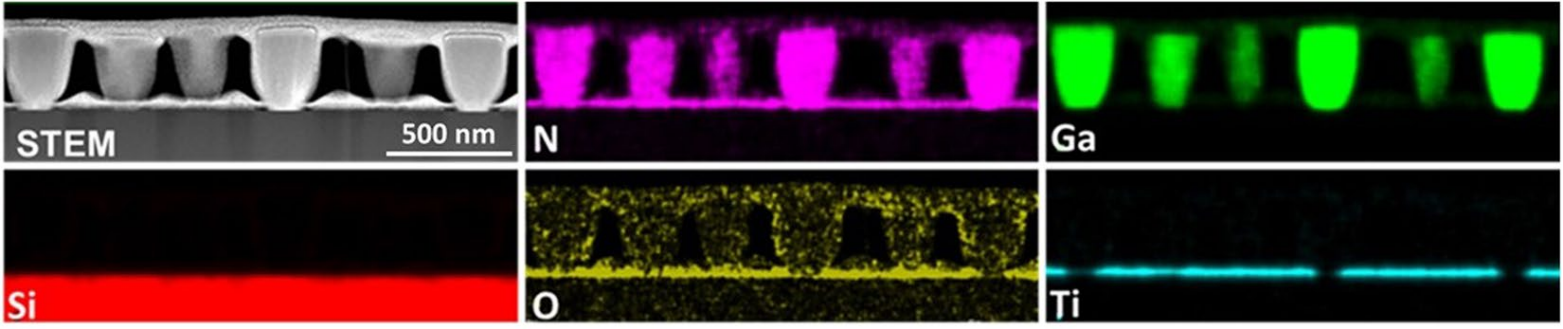
STEM-EDX elemental maps and corresponding STEM-HAADF overview of the 1 hour sample. The corresponding N, Ga, Si, O, and Ti elemental maps outline the GaN NRs, the TiN_x_ mask layer, and the SiO_x_ native layer at the interface with the Si substrate.

The structure and elemental distribution of the fully developed (1 hour) SAG GaN NRs was further investigated by TEM and STEM, as shown in Figs 5 and 6, respectively. The TEM analysis reveals that a small number of threading dislocations (Fig 5a and c) and stacking faults (Fig. 5b) are present in some, but not all (Fig. 5d), of the NRs and have the tendency to bend to the side-walls (Fig. 5). The vast majority of the defects originate from the lower part of the NRs. This is inferred to be caused by semi-coherent coalescence of the multiple nuclei grown on the native amorphous layer. The threading defects tend to be eliminated by mechanisms like bending towards surface facets (Fig. 5a) or termination in conjunction with stacking fault formation (Fig. 5b). The smaller diameter NRs contain fewer defects, which are either not present or are eliminated during the growth, in contrast to NRs exhibiting larger diameters. These observations indicate that high-crystal-quality GaN NRs without threading defects may be obtained by reducing the mask opening size, in order to achieve smaller diameter NRs.

The STEM-EDX elemental maps shown in Fig. 6 present an overview of the sample. The elemental maps show a uniform distribution of the Ga and N inside the NRs. It can be seen that the GaN grows exclusively inside the nano-openings. No apparent GaN nucleation is observed on the TiN_x_ mask even after 1 hour of growth. Intermixing between GaN, TiN, and Si is not observed, however, interdiffusion below the detection limit cannot be ruled out. The above results indicate that the Ga and N adatoms impinging on the TiN_x_ mask surface either migrated towards the growing NRs or desorbed from the surface. Selectivity is the combined effect of the chemistry of materials chosen for the growth and mask, which can be affected by the chemical potentials, growth temperature, partial pressure, mask thickness, etc. It has been shown that the selectivity was strongly affected by growth temperature and mask materials in SAG by MBE and MOCVD^19,30,31^. In our other experiments (not shown here), no obvious selectivity was observed at temperatures lower than 900 °C, and higher growth temperature is needed to achieve SAG using a thinner mask. Although both TiN_x_ mask and native SiO_x_ layer are amorphous, there is a noticeable thickness difference of 20 nm in comparison to 1–2 nm, respectively. The ultrathin SiO_x_ layer has much less effect on desorption of adatoms in comparison to TiN_x_, which results in a strong confinement for accommodating adatoms to form stable nuclei within nanoopenings.

### Growth mechanism

A key point in SAG is the suppression of nucleation on the mask. Considering this, the efficiency of SAG was found to be strongly correlated to adatom surface diffusion^16–19^. The surface diffusion length is mainly determined by temperature. When the surface diffusion of adatoms on the mask is too small, SAG fails and nucleation occurs on the mask. In the case of efficient SAG, the diffusion length is large enough that the adatoms impinging on the mask surface diffuse to the mask openings before nucleating. These adatoms are desorbed or migrate on the surface and get incorporated into the growing NR surface together with the adatoms that impinge on the opening surface.

**Figure 7:**
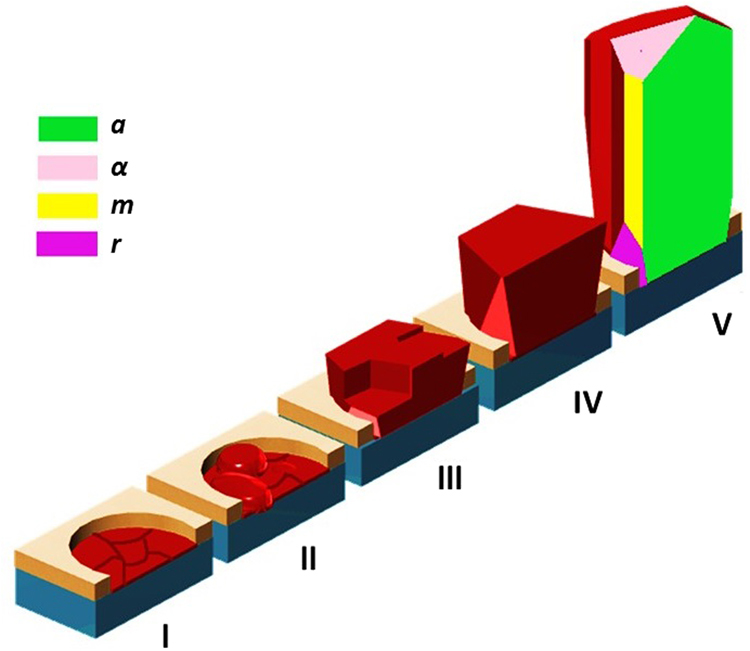
Schematic drawing showing the 5 growth stages that the GaN NRs undergo from the nucleation phase to the final NR shape. I- Wetting layer formation, II- 3D nuclei formation, III- Coarsening and coalescence of islands, IV- Single NR growth, V- Quasi-equilibrium crystal shape). The *r-*, *m-*, *a*-planes represent the indexed {$1\bar{1}0\bar{2}$}, {$1\bar{1}00$}, {$11\bar{2}0$} planes, while *α* can represent an *r*- or *s*-plane {$10\bar{1}1$}, or other high-indexed planes, depending on NR length.

In MSE, the formation mechanism of SAG-GaN NRs can be separated to five stages each of them having characteristic features (Fig. 7):

1^st^ stage – Wetting layer formation: The behavior of the initial GaN growth on the amorphous SiO_x_ layer is very different to both homoepitaxy of GaN on GaN substrate and heteroepitaxy of GaN on isostructural substrates in previous reports^5,19^. A thin polycrystalline wetting layer, approximately 1 nm thick is only formed inside the openings. This is a continuous layer that forms slowly in the first few minutes (Fig. 3b). The wetting layer is formed as a consequence of the confined growth area imposed by the mask. The formation of wetting layer on bare Si substrates was proposed to reduce interfacial energy, but it was not clearly observed to support this assumption^28,30^. By using a confined area and short growth time, the present observations show that the wetting layer is formed in the very beginning of the growth as an intermediate state.

2^nd^ stage – 3D nuclei formation: Beyond a critical thickness, the wetting layer growth proceeds in 3D, and nuclei are formed inside the openings. During the nuclei formation, multiple seeds are formed exclusively inside the openings. The 3D nuclei form on the wetting layer template, oriented with the *c*-axis parallel to the growth direction. Since *c*-axis growth is the energetically favored growth direction, adatoms diffuse inside the openings until they attach to the *c*-axis oriented nuclei. This behavior conducts to a random positional distribution of the nuclei inside the openings. The approximate lateral size of the nuclei is 20 nm (Fig. 2c). The stable nuclei grow in size giving rise to island formation. In addition, a high degree of *c*-axis oriented islands (verticality) with <5° tilting can be observed by measuring crystal planes’ angles, see Fig. 3, at different positions. The small tilting is related to the growth of GaN which follows and accommodates the roughness of the native oxide layer. This high verticality might be associated with the formation of dominant N-polar GaN nuclei grown at elevated temperatures on SiO_x_/Si by our MSE^24,27^. There are studies which relate GaN NRs with high verticality to nitrogen polarity, while Ga-polar nuclei often result in highly tilted NRs^32^. Also, SAG GaN NRs requires elevated growth temperatures leading to a preference for N-polar GaN which has a higher thermal stability^33^.

3^rd^ stage – Coarsening and coalescence of islands: At this stage no new nuclei are formed and the growth rate is higher than in the previous stages. The islands grow individually, at significant rates in both the vertical and lateral directions. At a height of approximately 70 nm, the islands coalesce and the growth continues of individual NRs (Fig. 2d,e). During coalescence, line defects such as threading dislocations (Fig. 5) are formed.

4^th^ stage – Single NR growth: After coalescence to single NRs, these grow vertically, exhibiting a flat surface. The growth along the semipolar direction is determined by the competition between *c*-axis growth and lateral diffusion of the adatoms from the TiN_x_ mask layer (Fig. 2f,g). Since the height of the NRs is still lower than the diffusion length of the adatoms impinging on the mask, in this stage lateral diffusion is significant, giving rise to the high energy semipolar planes.

5^th^ stage – Quasi-equilibrium crystal shape (Fig. 2h): After reaching a height of approximately 300 nm, the NR lateral growth is suppressed because the surface diffusion of adatoms on the mask and NR side walls is limited by the shadowing of incoming flux. By measuring the degree between the crystal planes after the complete coalescence of the islands forming single NRs, an extremely small deviation from verticality, of less than 1°, is obtained. Compared to the stage of nucleation, it can be seen that the verticality is further enhanced with coalescence and growth, with a negligible deviation in the single NRs. These NRs grow along the *c*-direction, with side walls consisting of both nonpolar planes: *a*- and *m*-planes. The reshaping of the NRs after 100 nm (Fig. 2f) where the initially developed lateral *a*-planes are suppressed can be observed. The NRs do not exhibit atomically sharp corners with coexistence of both nonpolar facets, (the *m*-planes and the *a*-planes). Until the coalescence of the islands is complete, the NRs grow exhibiting lateral *a*-planes. The top shape or the NRs evolves with their length. For example for shorter growth times, all NRs have a flat surface. For longer growth times, the top shape progressively becomes tapered in time – the inclination angle of the pencil-shaped top increases with NR length. This stage is driven by the energy minimization obtained by the morphological transition^5,34,35^. The existence of both facets was proven to act as diffusion channels for Ga atoms. The *m*-plane favors the lateral diffusion of the Ga adatoms, while when Ga adatoms arrive at the *a*-planes, diffusion along the *c*-axis is favored. Ga atoms then accumulate at the top of the crystal and axial growth continues^5,35^.

### Optical properties

**Figure 8:**
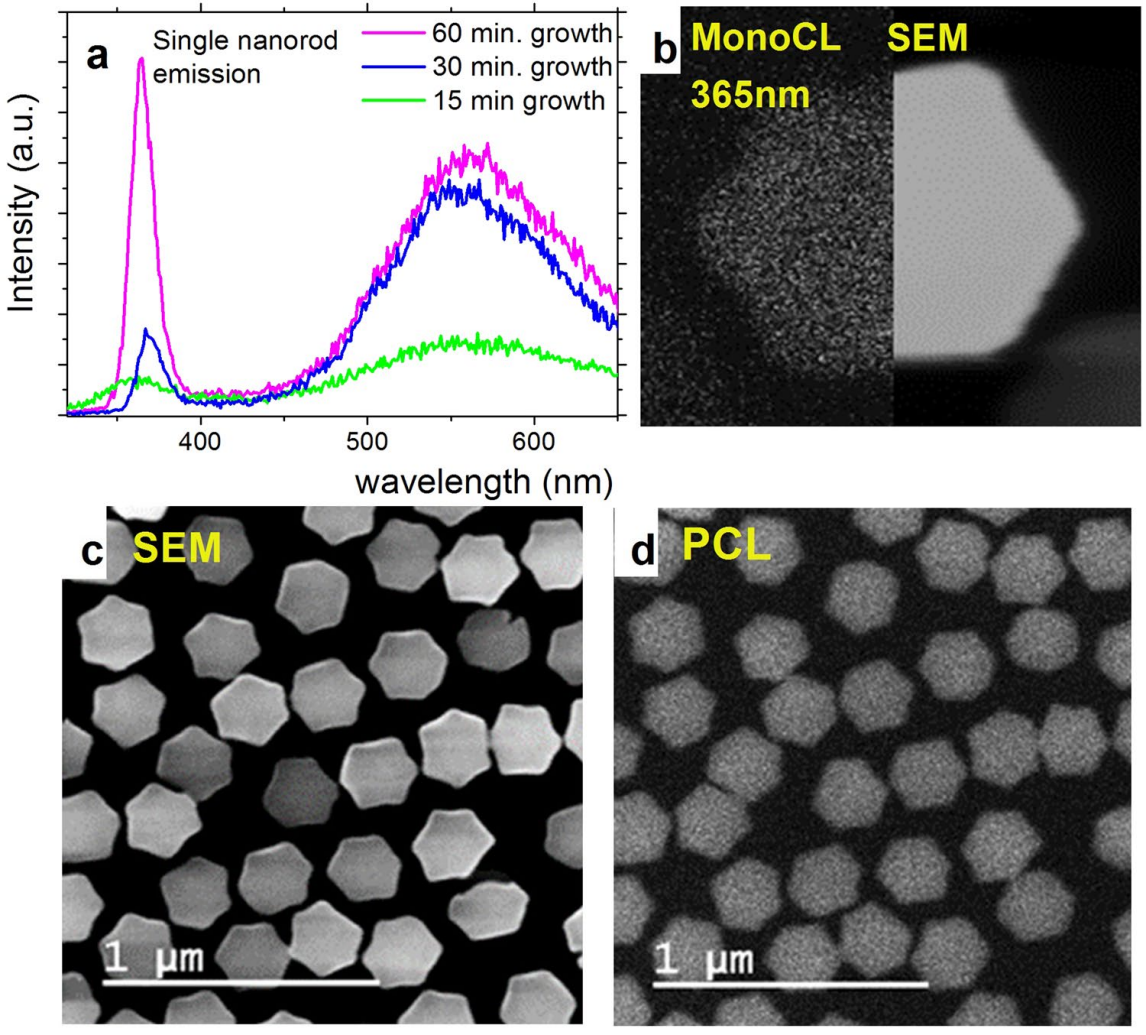
Optical properties of the SAG GaN NRs. (**a**) CL spectra of a single NR for different growth times – 15, 30, and 60 minutes. (**b**) Combined image between the bandedge emission of a single NR at 365 nm and the corresponding SEM image. (**c**) SEM overview of the sample grown for 1 hour and (**d**) the corresponding panchromatic image.

The cathodoluminescence (CL) spectra for single NRs and for different growth times are presented in Fig. 8a and reveals that the NRs exhibit strong bandedge emission. The GaN characteristic bandedge emission is centered at 365 nm. There is additionally a broad yellow band emission centered at approximately 565 nm. The origin of the yellow defect line is a debated topic^36–38^. There are two widely accepted mechanisms to describe it which include: 1) transition between a shallow donor and a deep acceptor or 2) a deep donor to a shallow acceptor recombination. Either way, this emission can be enhanced by the presence of impurities incorporated during growth. In the system, the main impurities are assumed to be oxygen and hydrocarbons coming from the gas injection system since we’ve observed a decrease of the yellow band emissions after doing gas purifying. As it can be seen, for a short growth time, the NRs possess weak luminescence. As the rod develops, the emission also increases, but is dominated by the yellow band emission, supposedly due to the defective initial growth and the presence of a higher number of dislocations. With increasing rod lengths, the defects are reduced and the bandedge emission becomes the most prominent.

A monochromatic CL image taken at 365 nm, from a single NR can be seen in Fig. 8b. The image reveals a characteristic bandedge emission with a strong and uniform intensity distribution inside the NR. The uniformity of the luminescence through the sample can be seen also in the panchromatic overview image presented in Fig. 8c and d. The NRs have similar intensity when compared with each other, which proves the uniformity of the sample.

The strong contribution from the yellow band in the shorter NRs can be explained by the comparably high contribution to the luminescence from the initial defective growth. The defective growth results in dislocation formation that can be easy paths for the diffusion of the point defects that contribute to the occurrence of this emission. For the longer NRs, the relative contribution from the defects is reduced due to the different dislocations annihilation, and the peak intensity of the bandedge emission becomes stronger than the yellow band emission.

### Lasing behavior

**Figure 9:**
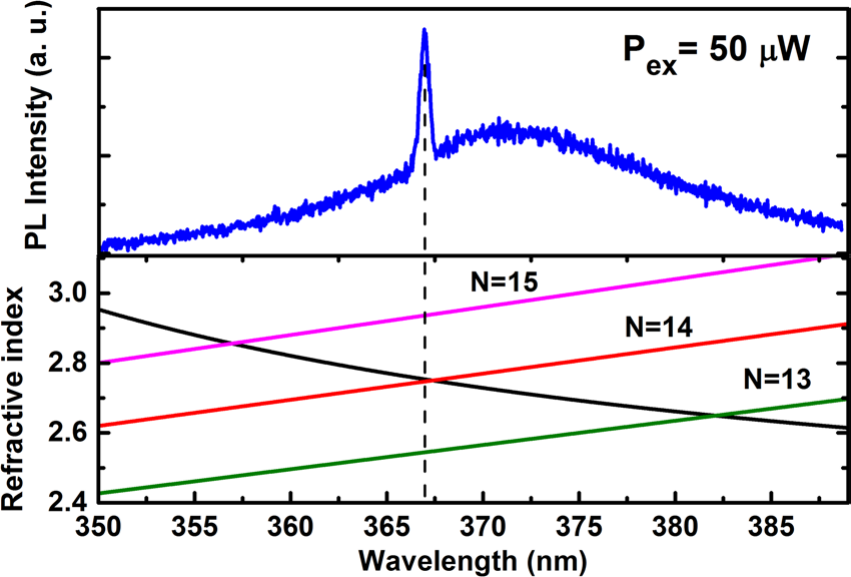
Lasing properties of GaN NRs: top shows the PL spectrum upon pulse laser excitation and the bottom plot displays the dispersion of wavelength dependent refractive index.

The top of Fig. 9 shows the lasing spectrum with the excitation power of 50 μW measured from the 2 hours grown sample. A broad spontaneous emission band located around 372 nm and a sharp peak at 367 nm are featured in the spectrum. The full width at half-maximum (FWHM) of the lasing mode is about 0.67 nm, so the Q factor is estimated to about 548 according to the equation Q = λ/Δλ, where λ and Δλ are the lasing mode wavelength and it’s FWHM, respectively. From the SEM images, we assume that the lasing cavity is of Fabry–Pérot (FP) type. The mode numbers of FP cavity can be numerically calculated by the following equation:

1 $$N=\frac{2nL}{{\lambda }_{FP}}$$

where *N* is longitudinal mode numbers of the FP cavity, ${\lambda }_{FP}$ is the wavelength of the lasing peak, L is the FP cavity length and n is the refractive index of the GaN NR. From the SEM results, the length of the GaN NRs and hence the FP cavity length, was estimated to about 937.4 nm. The refractive index of the GaN NRs dependent on wavelength is analyzed using a the first-order Sellmeier equation^39^:

2 $${n}^{2}=4.1+\,\frac{{\lambda }^{2}}{{\lambda }^{2}-0.096}$$

where A = 4.1 and B = 0.096 are the fitting parameters calculated from ref.^40^, and λ is in nm. Therefore, the refractive index of GaN NRs is about 2.75 calculated by the above mentioned equation.

As shown in the bottom of Fig. 9, the precise values for the FP lasing wavelengths can be determined at the intersections of a series of solid lines for different *N* from 13 to 15. When the mode number *N* = 14, the lasing wavelength is 367.6 nm, which is in agreement with the peak position shown in the top of Fig. 9, which confirms the FP as the lasing mechanism rather than random lasing or whispering gallery mode (WGM). Moreover, the resonance wavelengths of side modes (N = 14 and 15) are located at the tail of the emission spectrum, which makes lasing less feasible. Therefore, the single longitudinal mode lasing is achieved.

This process overcomes one of main challenges in synthesizing high-crystal-quality GaN films which arises from the lack of native substrates. The use of Si(001), represents an attractive option thanks to low cost, large wafer size and easy integration in optoelectronic devices, which combined with a relatively cheap method, like MSE, is highly beneficial. Furthermore, the disordered surface provided by the presence of the native SiO_x_ layer can later be applied on other substrates, like glass which presents the advantages of large-area processing and low manufacturing cost. The demonstrated lasing behavior achieved by optical pumping show high potential of using MSE-grown GaN NR based devices for the application in optical communication, optical interconnects, information processing, *etc*.

## Conclusions

We have reported on the initial growth stages and the time evolution of SAG GaN *c*-oriented NRs directly grown on Si(001) substrates by MSE. Additionally, these were investigated as to their structural and optical properties.

The growth of SAG NRs can be controlled with respect to both NR size and nucleation site in contrast to self-assembled NRs grown under identical conditions. An explicit 5-stage growth mechanism was proposed based on observations from samples grown for different sputtering times. The microstructural characterization of the same samples gave insights into the time evolution process, from the initial thin wetting layer to the well-defined, uniform, hexagonal NRs resulting from the coalescence of multiple initial nuclei. Formation of an energetically more stable geometry, a so-called quasi-equilibrium crystal shape, occurs after the complete coalescence of the islands.

Room-temperature CL spectroscopy of the SAG GaN NRs reveals a strong bandedge emission, and both monochromatic- and panchromatic-mode CL maps reveal uniform luminescence from the NRs. The bandedge emission increases with NR length, and is attributed to the relative reduction of structural defects originating from the incoherent coalescence of multiple nuclei during the initial stages. Additionally, a single-longitudinal-mode lasing spectrum measured on SAG NRs, pumped by a pulsed laser, was obtained at room temperature. The stimulated lasing behavior is attributed to the effect of Fabry–Pérot cavity, as a consequence of the well-faceted NR’s geometry.

## Experimental Methods

### GaN NRs growth

The growth of GaN NRs was performed by direct current (dc)-reactive MSE in an ultrahigh vacuum (UHV) chamber with a base pressure lower than 1 × 10^−8^ Torr and N_2_ (99,999999% pure) was used as the reactive working gas. A liquid Ga target (99.99999% pure) placed in a stainless steel crucible was used as a sputtering target. Details may be found elsewhere^24,27^.

**Figure 10:**
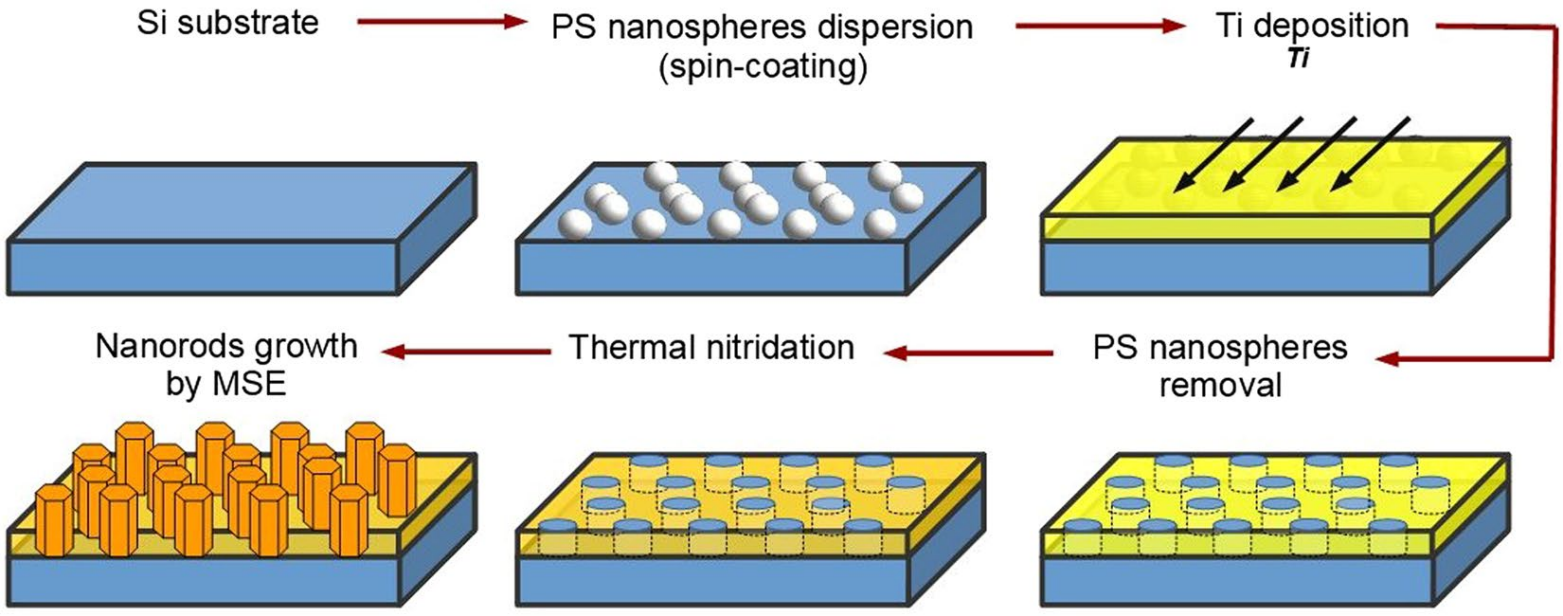
A schematic drawing of the steps performed in the NSL process.

Figure 10 schematically illustrates how the Si substrate was patterned by NSL with a TiN_x_ mask. A colloidal solution containing polystyrene nanospheres was first spread onto a Si substrate by spin-coating. Next, an approximately 20 nm thick Ti layer was deposited onto the substrate by e-beam evaporation. The nanospheres were then mechanically removed by tape-stripping. Subsequently, the Ti layer was thermally nitridized in an oven, in nitrogen atmosphere, at 850 °C for 2 h. Finally, the Si substrate, covered with a TiN mask exhibiting 150 nm sized openings, was subjected to GaN deposition by MSE.

Before the deposition, the Si substrates were degreased with trichloroethylene, acetone, and isopropanol in ultrasonic bath for 5 minutes each and blown dry with pure nitrogen. For eliminating possible contaminants, the substrates were outgassed for 60 minutes at 1000 °C before the growth in the MSE chamber. GaN NRs were grown on the patterned Si(001) substrates, at a temperature of 950 °C and N_2_ partial pressure of 20 mTorr. To obtain a time-lapse view of the growth mechanism, the deposition time was varied for otherwise identical growth conditions.

### Structural and compositional characterizations

Sample morphologies were characterized using a Zeiss Leo 1550 field-emission gun SEM. To elucidate the structural evolution of SAG-GaN NRs inside the TiN_x_ mask openings, the samples were tilted by 25 degrees away from substrate surface normal. The crystalline structures were analyzed by θ/2θ scan x-ray diffraction (XRD) using a Philips 1820 Bragg-Brentano diffractometer. Microstructural and elemental analysis were performed by high-resolution scanning transmission electron microscopy (STEM), as well as STEM-EDX mapping using the double-corrected Linköping FEI Titan^3^ 60–300. Cross-sectional samples for TEM analysis were prepared by focused ion beam (FIB) technique employing a Carl Zeiss Cross-Beam 1540 EsB system and following the procedure described elsewhere^41^.

### Optical characterizations

A Gatan MonoCL4 spectroscope equipped in the Zeiss Leo 1550 SEM was used for cathodoluminescence (CL) spectroscopy and mapping at an accelerating voltage of the electron beam of 10 kV. The emission from the samples was dispersed by a monochromator with a 150 lines/mm grating blazed at 500 nm and detected by a Peltier-cooled photomultiplier tube. 500 µm/500 µm entrance/exit slits were used for acquiring CL spectra. To obtain high-quality CL maps, fully opened slits were used in both monochromatic and panchromatic modes.

For the lasing spectrum measurement, a micro-photoluminescence (μ-PL) system was used, with a 266 nm Nd:YAG laser (pulse width of 550 ps and repetition rate of 8.73 kHz) as an excitation source. A 40 × UV objective lens was used to focus the laser beam and the emitted radiation was collected with the same objective lens. The emission spectra were recorded using a monochromator (Horiba iHR320) with a 1800 grooves/mm grating and a liquid-N_2_ cooled CCD array detector. All the measurements were performed at room temperature.

## Electronic supplementary material

Selective-area growth of single-crystal wurtzite GaN nanorods on SiO<sub>x</sub>/Si(001) substrates by reactive magnetron sputter epitaxy exhibiting single-mode lasing
