# Supplementary material for: Selective-area growth of single-crystal wurtzite GaN nanorods on SiOx/Si(001) substrates by reactive magnetron sputter epitaxy exhibiting single-mode lasing
Source: Sci Rep. 2017 Oct 5;7:12701. doi: 10.1038/s41598-017-12702-y (PMC5629253; doi:10.1038/s41598-017-12702-y)
Supplement: Supplementary file 1 — Selective-area growth of single-crystal wurtzite GaN nanorods on SiOx/Si(001) substrates by reactive magnetron sputter epitaxy exhibiting single-mode lasing [file 41598_2017_12702_MOESM1_ESM.pdf]

Selective-area growth of single-crystal wurtzite GaN nanorods on SiO_x/Si(001) substrates by reactive magnetron sputter epitaxy exhibiting single-mode lasing

*Elena Alexandra Serban[1], Justinas Palisaitis[1], Chia-Cheng Yeh[2], Hsu-Cheng Hsu[2], Yu-Lin Tsai[3], Hao-Chung Kuo[3], Muhammad Junaid[1], Lars Hultman[1], Per Ola Åke Persson[1], Jens Birch[1], Ching-Lien Hsiao[1]**

[1] Thin Films Physics Division, Department of Physics, Chemistry, and Biology (IFM), Linköping University, SE-581 83 Linköping, Sweden

[2]Department of Photonics, National Cheng Kung University, Tainan 701, Taiwan

[3]Department of Photonics and Institute of Electro-optical Engineering, National Chiao-Tung University, Hsinchu, Taiwan

Supplementary information

A semi-logarithmic plotted XRD $\theta/2\theta$ scan of the SAG-GaN NRs is shown in Figure S1. Except for the strong Si 002 (visible by dynamical diffraction) and 004 peaks located at 33.0° and 69.1° respectively, and the GaN 0002 and 0004 peaks located at 34.6° and 72.9° respectively, no other distinct peak is found.

The GaN diffraction peaks indicate that wurtzite GaN NRs were preferentially grown along 0001 direction. In the graph, marked by dotted lines are the reference positions for all hkil peaks of GaN in this range. As can be seen, only GaN 000l peaks are detected, which support these statements. Due to the very small thickness of the mask layer, the TiN_x is under the detection limit and no corresponding reflection is recorded.

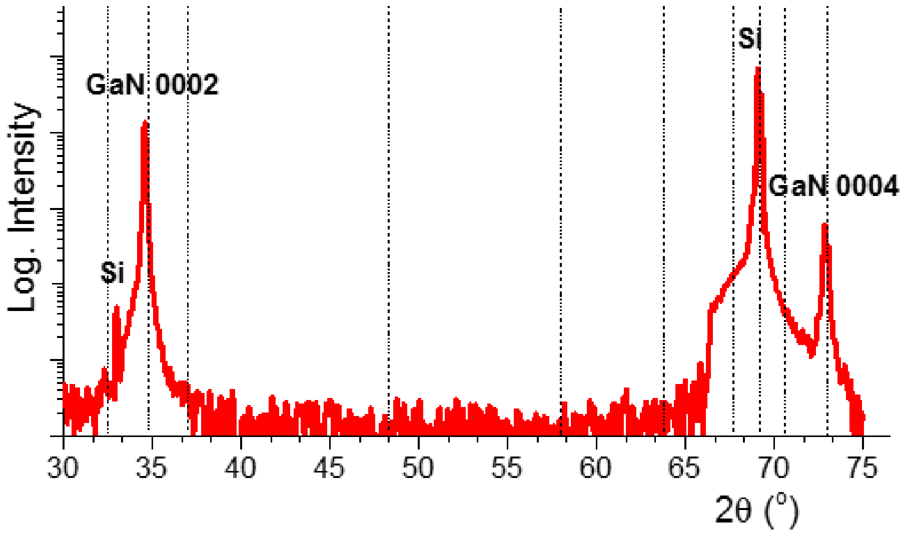

S 1. *XRD $\theta/2\theta$ long-range scan of the GaN NRs grown for 1 h. The dashed lines mark the peak positions for the Miller-Bravais indices for GaN*

In Figure S2 a statistical distribution representing the number of stable nuclei formed as a function of the opening size is presented. The data was obtained from the top-view SEM image of the sample grown for 5 minutes. As it can be seen, most of the openings exhibit sizes in between

145-165 nm and contain 4-5 stable nuclei that develop later on into a single NR. The area marked on the figure, represent the region with the characteristic average size of the openings. The formation of one nucleus is visible in one opening with a diameter of 117 nm. Opening diameters of more than 180 nm determines the formation of 6 or more stable nuclei, which will develop into one island. The formation of threading defects in larger diameter NRs, visible in the TEM images, is enhanced by the larger number of islands that coarsen and coalesce into the formation of the respective NR.

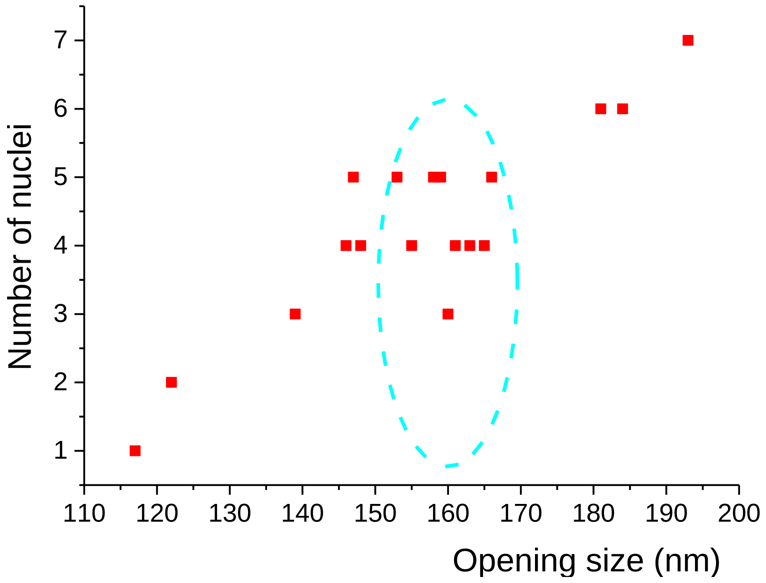

S 2. *Statistical distribution of the number of stable nuclei formed as a function of the opening size*

In the EDX line profile obtained in STEM though the interface $Si/SiO_x/TiN_x$ and the GaN nucleus shown in Figure S3, it can be seen that Ti and N signals follow together while O does not. The differentiation of N and Ti in EDX mapping is not possible in TiN due to complete overlap of the Ti-L peak with the N-K peak. The O-K peak does not follow the same trend resulting in a larger peak separation.

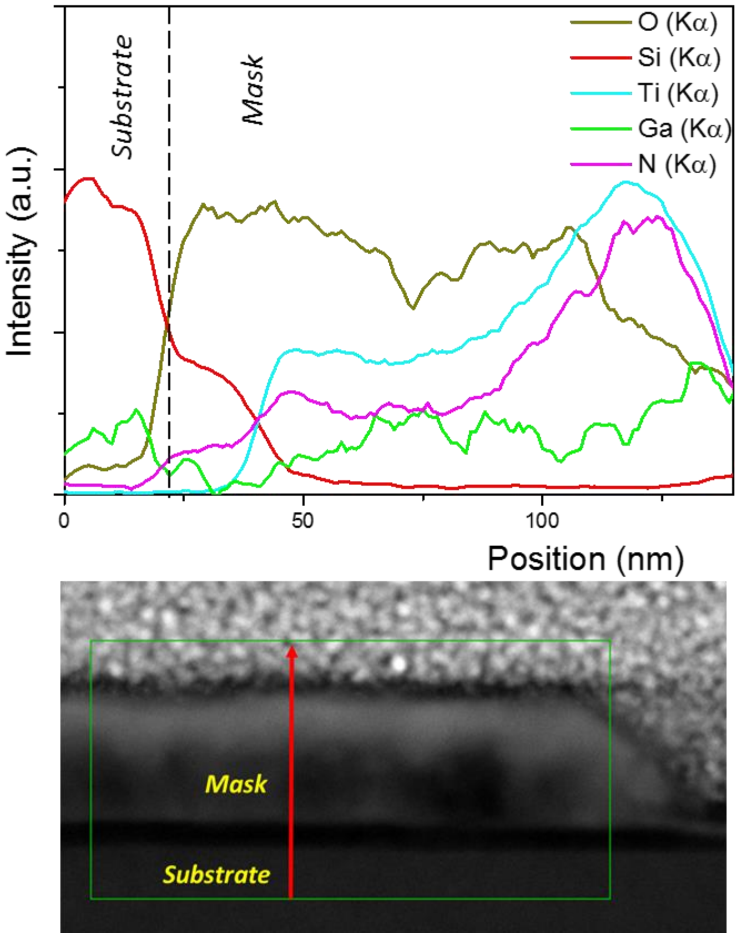

S 3. *EDX line profile taken at the interface of the Si substrate and* TiN_x *mask. The interface with the substrate is marked by dashed line.*

Reference:

S1. Gallium Nitride, PDF card number: 00-002-1078.
